# Depth-of-focus enhancement in optical coherence tomography via a cascaded image registration and fusion network for multi-focus imaging

**DOI:** 10.1117/1.JBO.31.7.076005

**Published:** 2026-07-28

**Authors:** Yuhui Chu, Sicheng Li, Huabing Tan, Mai Dan, Yunpeng Zhao, Pengpeng Zhao

**Affiliations:** a Binjiang Institute of Zhejiang University, Innovation Center for Smart Medical Technologies & Devices, Hangzhou, China; b Zhejiang University, College of Biomedical Engineering & Instrument Science, Hangzhou, China

**Keywords:** optical coherence tomography, depth-of-focus enhancement, cascaded image registration and fusion network, multi-focus images, electrically tunable lens, biomedical imaging

## Abstract

**Significance:**

Optical coherence tomography (OCT) is widely used in biomedical imaging, but its ability to clearly resolve fine structures is limited to a narrow depth of focus (DOF). This constraint restricts reliable visualization of tissue microstructures across extended depth ranges, making strategies that extend the DOF while preserving fine structural fidelity and image sharpness highly desirable.

**Aim:**

We aim to enhance the effective DOF of OCT imaging while preserving fine structural details and image sharpness by developing a deep-learning-based reconstruction framework for multi-focus OCT data.

**Approach:**

We developed a cascaded image registration and fusion network (CRFN) to process multi-focus OCT images acquired using a swept-source OCT system with dynamic focal modulation enabled by an electrically tunable lens. The proposed network consists of a registration module for spatial alignment of multi-focus images and a fusion module for focus map-guided reconstruction. CRFN operates in an unsupervised, training-free manner, in which the network parameters are optimized directly on the acquired multi-focus OCT images, without relying on large-scale pre-collected training datasets.

**Results:**

Experiments conducted on *ex vivo* and *in vivo* specimens demonstrate that the proposed method improves image quality compared with unfused and conventional multi-focus fusion approaches. Quantitative evaluations on *ex vivo* specimens show maximum improvements of 8.29 dB in signal-to-noise ratio and 1.56 dB in contrast-to-noise ratio, compared with conventional methods. Moreover, the effective DOF is extended by a factor of ∼2, enabling clearer visualization of fine structural details across an enlarged depth range.

**Conclusions:**

The proposed CRFN improves multi-focus OCT reconstruction quality and extends the effective DOF without increasing hardware complexity or relying on extensive training data, highlighting its robustness and potential generalizability for biomedical OCT imaging applications.

## 1 Introduction

Optical coherence tomography (OCT) is a non-invasive imaging modality capable of providing high-resolution, high-speed, and real-time volumetric visualization of biological tissues.^1^ These capabilities have established OCT as a cornerstone technology in biomedical diagnostics and clinical research.^2^^,^^3^ Nevertheless, OCT performance is fundamentally constrained by the inherent trade-off between depth of focus (DOF) and lateral resolution.^4^ Specifically, lateral resolution is inversely proportional to the numerical aperture (NA), whereas the DOF scales inversely with the square of the NA. Consequently, achieving higher lateral resolution inevitably shortens the DOF while achieving a sufficiently large DOF requires sacrificing lateral resolution. This intrinsic limitation has motivated the development of advanced strategies to mitigate defocus-induced degradation and improve image quality over an extended depth range.

Various approaches have been proposed to overcome this limitation. Computational refocusing techniques provide a versatile solution for DOF extension without hardware modifications, relying solely on post-processing algorithms. Representative methods include interferometric synthetic aperture (ISA),^5^^,^^6^ depth-encoded synthetic aperture (DESA),^7^ and multiple aperture synthesis (MAS).^8^^,^^9^ However, each exhibits intrinsic limitations: ISA demands strict phase stability for accurate inverse scattering reconstruction,^10^ DESA suffers from signal coupling loss caused by NA mismatch,^11^ and MAS involves a compromise in scanning speed.^8^ Beyond computational refocusing, beam engineering offers another route to DOF extension by tailoring the optical field. Techniques such as Bessel beams,^12^ Airy beams,^13^ needle-shaped beams,^14^^,^^15^ multi-level diffractive beams,^16^ and multifocal beams^17^ have been explored to manipulate light propagation for extending DOF or generate multiple focal planes. Despite their effectiveness, these methods are challenged by reduced signal-to-noise ratio (SNR), increased optical complexity, and restricted adaptability in practical OCT systems.

Given the constraints of computational refocusing and beam-engineering approaches, focal plane modulation has emerged as a practical and adaptable solution for extending DOF. Traditional implementations rely on mechanical focus scanning,^18^ whereas recent systems employ electrically tunable lenses (ETLs). Compared with mechanical scanning, ETLs offer millisecond-level response and settling times, enabling vibration-free axial focal shifts through current-controlled curvature modulation.^19^^,^^20^ Despite these advantages, ETL-based DOF extension still faces several challenges. Multi-focus OCT acquisition combined with image fusion techniques is widely used to improve robustness and applicability.^21^^–^^23^ Conventional methods typically suppress out-of-focus regions using Gaussian windows, followed by image registration and weighted averaging to generate fused results.^21^ However, these results often exhibit defective pixels, partial loss of fine microstructural details, and increased sensitivity to noise, which collectively undermine continuity and structural fidelity. Alternatively, an adaptive focus tracking strategy dynamically adjusts the ETL driving current to match the focal plane with nearly each A-scan according to the known sample contour,^24^^–^^26^ enabling single B-scan reconstruction without multi-image stitching. Although this strategy improves acquisition efficiency, it depends heavily on accurate contour estimation and precise synchronization between OCT scanning and ETL modulation. Any misalignment can lead to focal mismatches and geometric distortion, limiting its robustness in practical imaging scenarios.

The remarkable success of deep learning (DL) in OCT image enhancement motivates its application to multi-focus image registration and fusion. However, most existing DL models are supervised and require large, annotated datasets, which are challenging to acquire in practice. Recently, the deep image prior (DIP) paradigm demonstrated that the intrinsic structure of a deep network can capture the statistical properties of input images as an implicit prior,^27^^,^^28^ enabling low-level tasks without large-scale training datasets. In this paradigm, the network parameters are optimized at test time for each individual input, eliminating the need for pretraining. This property makes it particularly attractive for OCT applications where paired ground-truth data are unavailable. Motivated by this, we adopt a test-time optimization strategy and extend it to jointly address multi-focus image registration and fusion in OCT imaging.

In this study, we present an approach for DOF enhancement in OCT imaging via a DL-based image reconstruction coupled with ETL-enabled multi-focus acquisition. A swept-source OCT (SS-OCT) system incorporating an ETL is first implemented to modulate the focal plane and acquire multi-focus OCT images, following established dynamic focus OCT strategies. Building upon this multi-focus acquisition, the primary contribution of this work lies in the development of a cascaded image registration and fusion network (CRFN) designed to address the misalignment and reconstruction challenges inherent to multi-focus OCT imaging. The proposed CRFN adopts a two-stage architecture consisting of a registration module and a fusion module. The registration module combines a displacement field predictor network (DFPN) and a spatial transformer network (STN) to achieve accurate spatial alignment and geometric correction of multi-focus images. The aligned images are subsequently processed by the fusion module, which integrates a focus map estimation network (FMEN) with an image fusion network (IFN) to selectively combine complementary in-focus information across depths. Unlike conventional supervised DL approaches, this network operates in an unsupervised, self-optimization manner, iteratively updating its parameters directly from the input images, without requiring pre-trained models or training datasets. Experimental validations on *ex vivo* specimens across multiple tissue types (porcine eyes, porcine muscles, and chicken wings), along with *in vivo* volumetric imaging in a mouse model, demonstrate that the proposed CRFN achieves an approximately twofold extension of the effective DOF relative to input source images. Moreover, it consistently outperforms classical approaches in terms of image sharpness, structural fidelity, and quantitative metrics.

## 2 Methods

### 2.1 ETL-Incorporated SS-OCT System

A customized SS-OCT system integrated with an ETL was developed to enable dynamic focal plane modulation, as shown in Fig. S1 in the Supplementary Material. The ETL is incorporated into the sample arm to allow rapid adjustment of the focal position, thereby enabling multi-focus acquisition. By synchronizing the ETL modulation with the scanning process, B-scan images at different focal positions can be sequentially acquired, providing the basis for subsequent multi-focus registration and fusion. Detailed optical configuration and system synchronization are described in the Supplementary Material.

### 2.2 Focal Plane Modulation Characteristics of the ETL-Equipped Sample Arm

#### 2.2.1 Ray-tracing simulations and experimental validation

The integrated optical assembly, comprising ETL, dual-axis galvanometer scanner (DGS), and achromatic doublet (AD), was modeled and optimized using a ray-tracing platform. Simulations were performed at a central wavelength of 1310 nm with a 3-mm entrance pupil, matching the collimated beam in the system. The focal power (FP) of the ETL was varied from 0 to +10 diopters (dpt), covering the full positive tuning range. The resulting curvature radius (R) of the deformable surface was calculated as

$$R=\frac{n_{\mathrm{ETL}}-1}{\mathrm{FP}},$$ (1)

where $n_{\mathrm{ETL}}$ denotes the refractive index of the ETL (≈1.30), and R is expressed in meters. A higher FP corresponds to a smaller R, yielding a more convex liquid surface and enhanced beam focusing capability. Within the DGS, the focused beam initially propagated along the x-axis and then underwent two sequential reflections at the x- and y-axis mirrors. These reflections reoriented the beam toward the z-axis, after which it was directed to downstream AD. The used AD in the model was specified to have a focal length of 25 mm and a clear aperture of 12.70 mm.

**Fig. 1:**
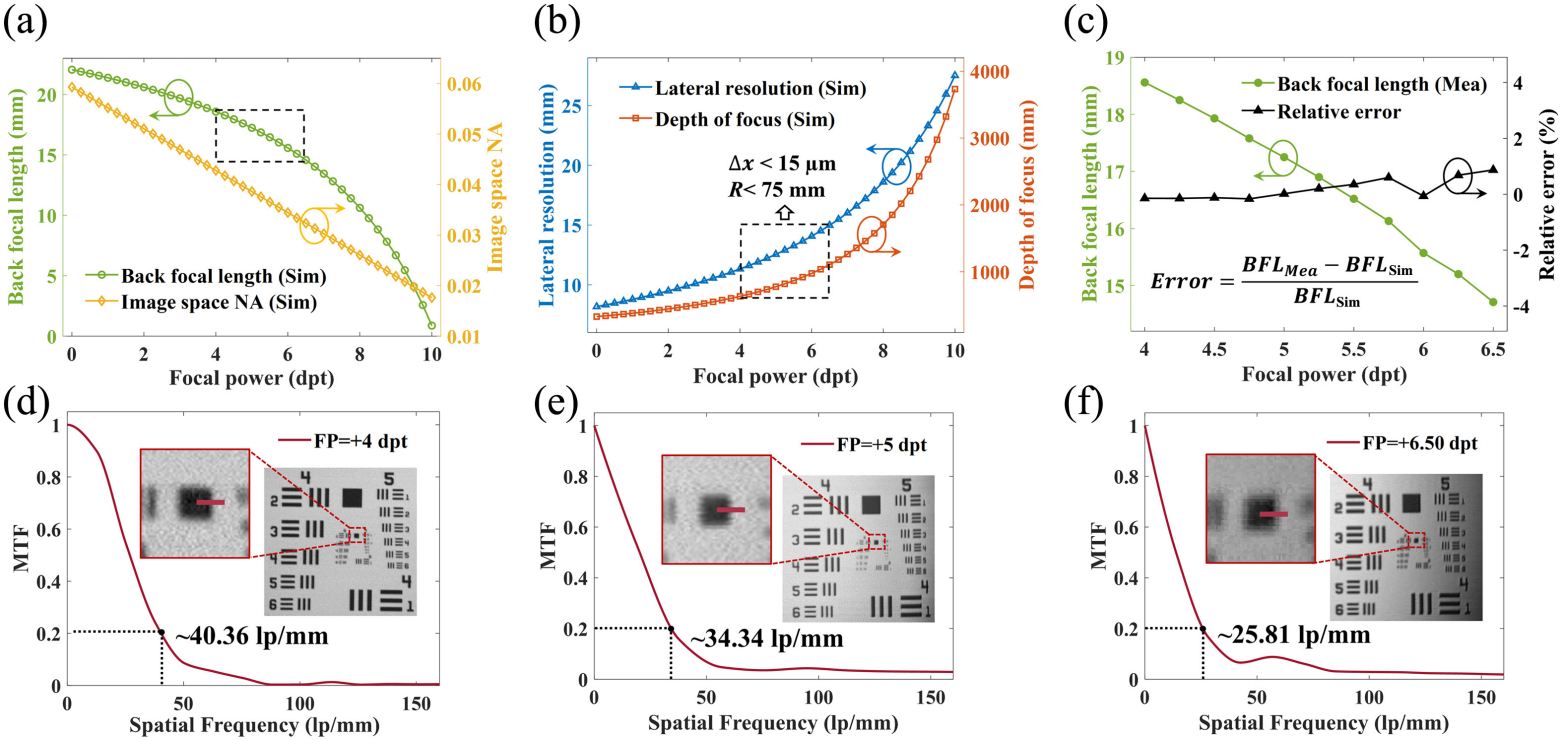
Ray-tracing simulations and experimental validation of focal plane modulation in the ETL-equipped sample arm. (a) Simulated BFL and image space NA as functions of focal power. (b) Calculated lateral resolution and DOF derived from Eqs. (2) and (3) (in air). (c) Measured BFL agrees with the simulations, with a relative error within ±1%. (d)–(f) MTF curves extracted along the red lines from the *en face* OCT images of a USAF-1951 resolution target at the focal power of +4, 5, and 6.50 dpt, respectively. The black dotted rectangles in panels (a) and (b) represent the experimentally used focal power range (+4 to +6.50  dpt).

To characterize the imaging performance and focal plane tuning behavior of the integrated assembly, key parameters including the back focal length (BFL), image space NA, lateral resolution, and DOF were evaluated across the entire positive ETL tuning range. As shown in Fig. 1(a), the BFL decreases monotonically as the FP increases from 0 to +10  dpt, producing a focal plane shift of ∼21  mm. The image space NA also decreases monotonically with increasing FP, which alters the lateral resolution and DOF in OCT imaging [Fig. 1(b)] according to^29^

$$\Delta x=0.37\frac{\lambda_{0}}{\sin(\theta )}=0.37\frac{n_{0}\lambda_{0}}{NA},$$ (2)

$$d_{\mathrm{DOF}}=0.221\frac{\lambda_{0}}{\sin^{2}(\frac{\theta }{2})}=0.221\frac{\lambda_{0}}{\sin^{2}[\frac{\sin^{-1}(\frac{NA}{n_{0}})}{2}]},$$ (3)

where $\lambda_{0}$, θ, and $n_{0}$ denote the working wavelength, half the angular aperture, and the refractive index of the medium, respectively.

Although the simulations covered the full 0 to +10  dpt range, the experimentally usable range is more restricted. At low focal powers (corresponding to a large curvature radius), the ETL is more susceptible to gravity-induced asymmetric deformation of the liquid interface, leading to increased optical aberrations, particularly coma and astigmatism, which degrade imaging performance.^30^ To mitigate aberration-prone regime, the experimentally used focal power range was empirically restricted to +4 to +6.50  dpt, corresponding to R<75  mm [Eq. (1)] and Δx<15  μm [Eq. (2)]. Within this range, the measured BFL [green solid line with filled circular markers in Fig. 1(c)] agrees with the simulated values [Fig. 1(a)], with a relative deviation below ±1% [black solid line with filled triangular markers in Fig. 1(c)], validating the accuracy of the optical model and the reliability of the focal tuning mechanism.

The focus-dependent lateral resolving capability of the OCT system was further validated using the knife-edge method by acquiring an *en face* OCT image of a USAF-1951 resolution target positioned at focal power of 4, 5, and 6.5 dpt, as shown in Figs. 1(d)–1(f), respectively. The modulation transfer function (MTF) at a modulation of 0.2 is ∼40.36  lp/mm (12.39  μm) at 4 dpt, 34.34  lp/mm (14.56  μm) at +5  dpt, and 25.81  lp/mm (19.37  μm) at +6.5  dpt, consistent with the trend observed in Fig. 1(b). Lateral spatial calibration also confirmed a linear relationship between the DGS driving voltage and the lateral field of view, with a slope of ∼1.10  mm/V.

To evaluate the impact of chromatic aberration across the swept-source bandwidth on the axial point spread function (PSF), an axial resolution at a focal power of +6.5  dpt was measured using a mirror target under two conditions: without and with the ETL in the optical path. As shown in Fig. S2 in the Supplementary Material, the normalized axial resolution across the full imaging depth (5 mm in air for the employed swept-source laser) exhibits negligible difference between the two cases (17.66 versus 18.40  μm), indicating that the ETL does not introduce measurable degradation of the axial PSF within the operating wavelength range. Therefore, the effect of ETL-induced chromatic aberration on axial imaging performance is considered negligible in the proposed system.

#### 2.2.2 Focal power reproducibility of ETL

The response time of the TEL is ∼5  ms, while the settling time is ∼40  ms. To ensure stable imaging, a waiting interval exceeding 40 ms was introduced among consecutive focal power steps. Consequently, each B-scan was acquired after the ETL had reached a stable state, ensured high B-scan-to-B-scan focal reproducibility, and minimized geometric distortion.

To evaluate focal power reproducibility, repeated B-scan acquisitions (N=50) were performed at the same focal power (+6.5  dpt), with a mirror target positioned at different depths. For each B-scan, axial peak positions corresponding to different depths were extracted, and both the interquartile range (IQR) and the standard deviation (SD) were computed to quantify focal stability. As shown in Fig. S3 in the Supplementary Material, the narrow IQRs across four representative depths (<2.38  μm) indicate consistently small variations in axial peak positions. In addition, the SDs across these depths remain below 1.39  μm, which is significantly smaller than the system axial resolution, confirming stable focal power modulation and high reproducibility across repeated acquisitions.

#### 2.2.3 Axial optical path difference shift induced by ETL focal tuning

To evaluate the potential axial optical path difference (OPD) variation induced by the ETL, we performed an experimental calibration using a mirror target placed at two fixed image depths. OCT B-scan images were acquired while varying the ETL focal power from +4 to +6.50  dpt while keeping all other settings unchanged. The axial peak position was extracted from the B-scan for each focal power, enabling a quantitative characterization of the apparent depth shift as a function of ETL tuning.

As shown in Fig. S4 in the Supplementary Material, the measured axial positions increase monotonically with increasing focal power. A redshift of ∼99.43  μm is observed at a depth of ∼1.5  mm when tuning the focal power from +4.0 to +6.50  dpt, while a similar redshift of ∼104.10  μm is measured at ∼3.5  mm, which can be effectively compensated by the subsequent registration module in the proposed framework.

Collectively, the simulations and experimental results demonstrate that the limited focal range of a single focus setting prevents consistent maintenance of fine structural details and image sharpness across the full ∼5-mm imaging depth. In addition, focal tuning introduces axial OPD shifts that further induce geometric distortions in B-scan images acquired at different focal powers. These limitations highlight the need for multi-focus OCT image registration and fusion strategies to achieve high-quality imaging over the entire depth range.

### 2.3 Proposed CRFN for Multi-Focus OCT Image Registration and Fusion

**Fig. 2:**
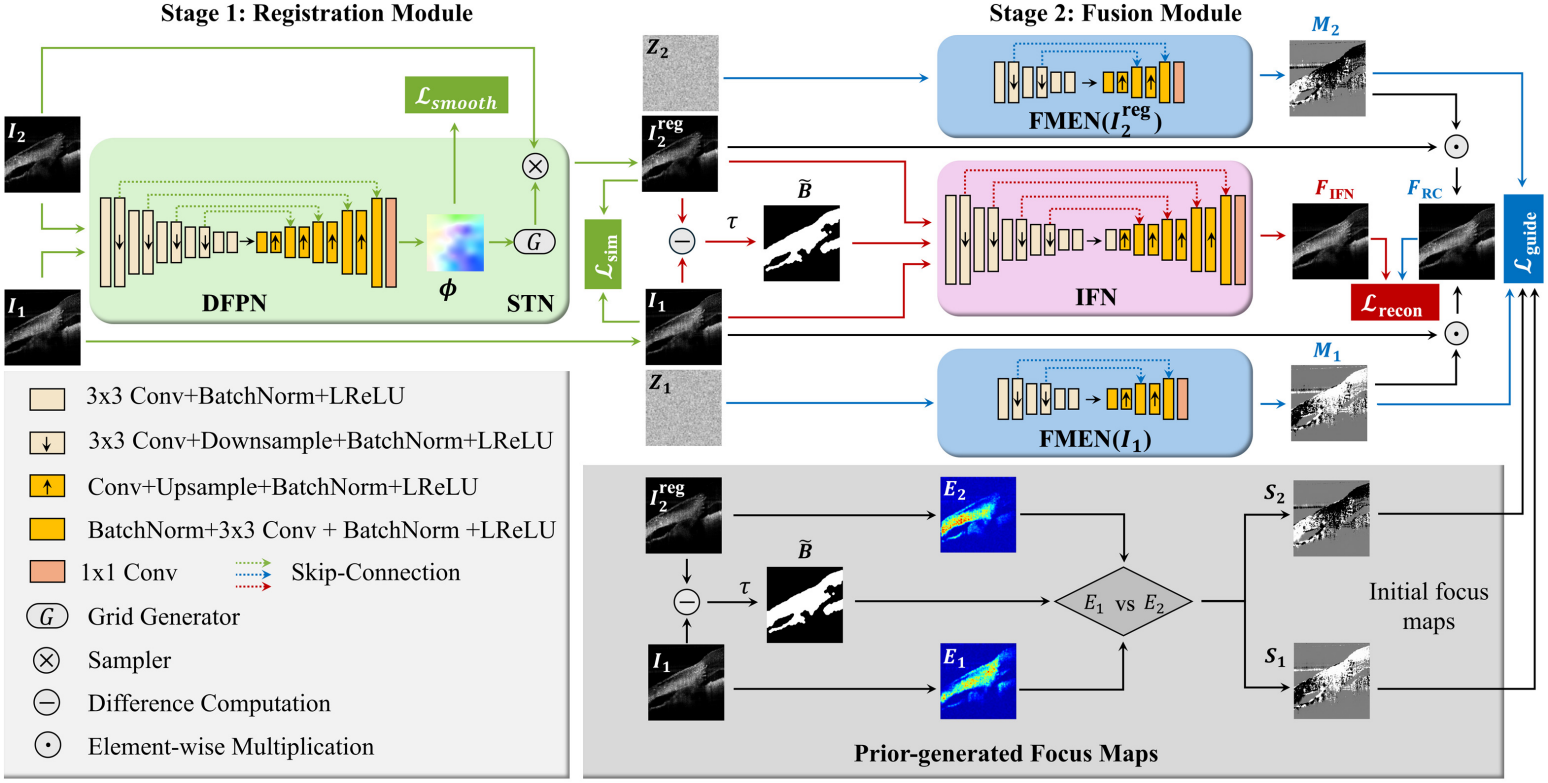
Overview of the proposed CRFN framework. Source images ($I_{1}$ and $I_{2}$) are first aligned by the registration module, producing the registered image ($I_{2}^{\mathrm{reg}}$) for the downstream fusion module. The FMEN predicts focus maps $M_{1}$ and $M_{2}$ for $I_{1}$ and $I_{2}^{\mathrm{reg}}$, which are guided by the prior-generated focus cues $S_{1}$ and $S_{2}$ ($L_{\text{guide}}$), and directly influence the reconstruction of $F_{\mathrm{RC}}$ ($F_{\mathrm{RC}}=I_{1}⨀M_{1}+I_{2}^{\mathrm{reg}}⨀M_{2}$). The IFN takes $I_{1}$, $I_{2}^{\mathrm{reg}}$, and $\tilde{B}$ as input and generates the fused image, which is tightly coupled with the FMEN through a reconstruction constraint ($L_{\mathrm{recon}}$).

To achieve accurate registration and high-quality fusion of multi-focus OCT images, we propose an unsupervised DL framework termed CRFN. The overall architecture, shown in Fig. 2, consists of two cascaded yet functionally coupled stages: a registration module (stage 1) and a fusion module (stage 2). Given multi-focus OCT B-scans acquired at different ETL focal powers, the registration module first estimates dense non-rigid deformation fields to correct spatial misalignments. The geometrically aligned images are then processed by the fusion module, which integrates complementary in-focus information across depths to generate an all-in-focus reconstruction with extended DOF.

#### 2.3.1 Stage 1: registration module

B-scan images acquired at different focal planes exhibit complicated non-rigid geometric deformations, which cannot be adequately corrected using rigid or affine transforms. Without proper alignment, these inconsistencies propagate into the subsequent fusion stage, inevitably leading to structural inconsistencies and artifacts. To address this issue, accurate registration is therefore a critical step. The registration module integrates a DFPN with an STN to achieve accurate pixel-wise alignment.

The DFPN adopts a U-Net-like encoder–decoder architecture designed to capture multi-level features and reconstruct nonlinear displacement fields that explicitly model the non-rigid deformations between input image pair ($I_{1}$ and $I_{2}$). The architecture comprises five hierarchical resolution levels, with feature extraction performed at each level. The encoder consists of four downsampling operations, each including a convolutional layer (Conv), batch normalization (BatchNorm), and a leaky rectified linear unit activation, progressively reducing the spatial resolution. The decoder mirrors this structure with four upsampling operations to restore the spatial resolution. Skip connections concatenate these encoder and decoder features to preserve fine structural information during displacement estimation. The DFPN outputs a dense displacement field ϕ, generating the pixel-wise transformation required for precise alignment.

The STN, which consists of a grid generator and a sampler, employs the estimated ϕ to warp $I_{2}$. For each pixel p=(j,k) in ϕ, the grid generator computes the pixel location $p^{\prime }=p+\phi$ in $I_{2}$. The sampler then performs differentiable bilinear interpolation to estimate pixel intensities at non-integer $p^{\prime }$, yielding the registered image,

$$I_{2}^{\mathrm{reg}}=I_{2}\circ \phi (p)=\underset{q\in Z\left(p^{\prime }\right)}{\sum }I_{2}(q)\underset{d\in \left\{j,k\right\}}{\prod }(1-|p_{d}^{\prime }-q_{d}|),$$ (4)

where $Z(p^{\prime })$ represents the set of neighboring pixels around $p^{\prime }$, and the product term ∏(·) aggregates coordinate-wise interpolation weights. This interpolation enables precise pixel-level warping, which is essential in OCT imaging where minor misalignments can degrade fine structural details.

#### 2.3.2 Stage 2: fusion module

Multi-focus OCT image fusion can be formulated as a spatially weighted reconstruction problem, where the fused image is obtained by selectively combining image pair according to their focus-dependent contributions. However, directly estimating accurate focus maps in OCT images is challenging due to the presence of speckle noise, low-contrast regions, and background-dominated areas. Simple weighted averaging often results in loss of fine structural information and blurring of edges. Moreover, OCT images differ fundamentally from natural images in that they contain not only focused and defocused sample regions but also significant background regions with low structural relevance.

To address these challenges, we design the fusion module as a joint optimization framework that simultaneously estimates the focus maps and reconstructs the fused image under implicit deep priors. Specifically, the module consists of two coupled subnetworks: an FMEN which predicts spatial focus maps under an implicit constraint and an IFN which models the image prior to reconstruct the fused image.

The FMEN predicts focus maps for $I_{1}$ and $I_{2}^{\mathrm{reg}}$ using a lightweight encoder–decoder architecture. It comprises three hierarchical resolution levels, with feature extraction performed at each level. The encoder includes two downsampling operations, and the decoder mirrors this structure with two upsampling operations to restore spatial resolution. The FMEN takes random noise inputs $Z_{1}$ and $Z_{2}$ and predicts corresponding spatial focus maps $M_{1}$ and $M_{2}$ for $I_{1}$ and $I_{2}^{\mathrm{reg}}$, respectively, following the deep image prior paradigm,

$$M_{1}=G_{\mathrm{FMEN}}(Z_{1})\;M_{2}=G_{\mathrm{FMEN}}(Z_{2}),$$ (5)

where $G_{\mathrm{FMEN}}$ represents the FMEN. Here, $Z_{1}$ and $Z_{2}$ randomly sampled from a uniform distribution U(0,0.1), where the upper bound 0.1 was chosen to keep the initial input magnitude small relative to the normalized image intensity range [0, 1]. This ensures the networks begin optimization from a near-zero state, consistent with the implicit regularization properties of the DIP framework.

To guide the focus map estimation within the DIP-based FMEN, we construct prior-generated focus cues $S_{1}$ and $S_{2}$ to provide explicit supervision on spatial focus distribution, thereby stabilizing the optimization and preventing ambiguous or diffuse focus predictions. The difference map-based binary mask $\tilde{B}$ is innovatively introduced to separate the foreground sample region from the background, tailoring to OCT-specific structural and intensity characteristics. This prior $\tilde{B}$ is obtained by thresholding the intensity difference between $I_{1}$ and $I_{2}^{\mathrm{reg}}$, which is inspired by a standard remote sensing image detection method,^31^

$$\overset{˜}{B}(j,k)=\{\begin{array}{cc} 1, & \text{if }|I_{1}(j,k)-I_{2}^{\mathrm{reg}}(j,k)|>\tau \\ 0, & \text{if }|I_{1}(j,k)-I_{2}^{\mathrm{reg}}(j,k)|\leq \tau \end{array},$$ (6)

where (j,k) represents the pixel location. Here, the threshold τ, which depends on the background region’s intensity in the normalized source images, was designed to separate the sample region from low-variation background regions. This design suppresses low-variation background regions that are irrelevant for focus estimation. With the foreground sample regions ($\overset{˜}{B}=1$), pixel-wise sharpness maps of $I_{1}$ and $I_{2}^{\mathrm{reg}}$ ($E_{1}$ and $E_{2}$) are evaluated using a local energy measure, which captures more high-frequency structural variations and yields higher local energy values in the regions in sharp focus,^32^

$$E_{1}(j,k)=\overset{r}{\underset{v=-r}{\sum }}\overset{r}{\underset{\varpi =-r}{\sum }}{\left[I_{1}(j+v,k+\varpi )\right]}^{2}\;E_{2}(j,k)=\overset{r}{\underset{v=-r}{\sum }}\overset{r}{\underset{\varpi =-r}{\sum }}{\left[I_{2}^{\mathrm{reg}}(j+v,k+\varpi )\right]}^{2},$$ (7)

where the summation is performed over a square window of size l×l (r=3 and l=7) centered at pixel coordinates (j,k). (v,ϖ) denotes the relative offset coordinates within the local neighborhood. The prior focus maps $S_{1}$ and $S_{2}$ are then constructed by comparing local energy,

$$\begin{array}{c} S_{1}(j,k)=\left\{ \begin{array}{ll} 1 & \text{if }\overset{˜}{B}(j,k)=1\text{ and }E_{1}(j,k)\geq E_{2}(j,k)\\ 0 & \text{if }\overset{˜}{B}(j,k)=1\text{ and }E_{1}(j,k)<E_{2}(j,k)\\ 0.5 & \text{if }\overset{˜}{B}(j,k)=0 \end{array}\right. \end{array}\;S_{2}(j,k)=\left\{ \begin{array}{ll} 1 & \text{if }\overset{˜}{B}(j,k)=1\text{ and }E_{1}(j,k)<E_{2}(j,k)\\ 0 & \text{if }\overset{˜}{B}(j,k)=1\text{ and }E_{1}(j,k)\geq E_{2}(j,k)\\ 0.5 & \text{if }\overset{˜}{B}(j,k)=0 \end{array}\right.,$$ (8)

where pixels in the foreground sample regions are assigned based on relative sharpness, while background regions ($\overset{˜}{B}=0$) are assigned a neutral value (0.5) to avoid introducing unreliable supervision. This design yields a structure-aware and noise-robust focus prior, which effectively stabilizes the estimation of focus maps and prevents ambiguous or diffused attention during optimization.

The IFN, which adopts an architecture such as the DFPN, takes $I_{1}$, $I_{2}^{\mathrm{reg}}$, and $\overset{˜}{B}$ as input and generates the fused image,

$$F_{\mathrm{IFN}}=G_{\mathrm{IFN}}[\text{concat}(I_{1},I_{2}^{\mathrm{reg}},\overset{˜}{B})],$$ (9)

where $G_{\mathrm{IFN}}$ represents the IFN, and concat(·) refers to the concatenation operation along the channel dimension.^28^ Unlike conventional fusion networks, IFN does not operate independently but is tightly coupled with FMEN through a reconstruction constraint,

$$F_{\mathrm{RC}}=I_{1}⊙M_{1}+I_{2}^{\mathrm{reg}}⊙M_{2},$$ (10)

where ⊙ represents the element-wise multiplication. This formulation enforces that the fused image is physically consistent with the input images and their corresponding estimated focus maps, preventing hallucinated structures and ensuring interpretability.

Unlike sequential pipelines, FMEN and IFN are optimized jointly in a self-supervised manner. Specifically, FMEN provides spatial decomposition (where to take information), while IFN enforces reconstruction consistency (how to combine information). This mutual constraint mechanism ensures that erroneous focus maps are penalized through reconstruction inconsistency and blurred, or artifact-prone fused images are corrected via mask refinement. This joint optimization framework unifies focus map estimation and image fusion into a mutually constrained optimization framework, enabling robust multi-focus OCT fusion without requiring ground-truth supervision or pre-training, which is particularly attractive for OCT applications where paired ground-truth data and large training dataset are unavailable.

#### 2.3.3 Loss function

The loss function of the registration module $L_{\mathrm{reg}}$ consists of two parts: a similarity term $L_{\mathrm{sim}}$, which is the mean squared error (MSE) between $I_{1}$ and $I_{2}^{\mathrm{reg}}$, and a smoothness regularization term $L_{\text{smooth}}$, which penalizes large spatial variations in the deformation field $\vec{u}$, thereby encouraging smooth and physically plausible deformations. The $L_{\mathrm{reg}}$ are defined as follows:^33^

$$L_{\mathrm{reg}}=L_{\mathrm{sim}}+\theta L_{\text{smooth}}=\frac{1}{\left|T\right|}\underset{p\in T}{\sum }{\left[I_{2}^{\mathrm{reg}}(p)-I_{1}(p)\right]}^{2}+\theta \underset{p\in T}{\sum }{\left\|\nabla \overset{\to }{u}\left(p\right)\right\|}^{2},$$ (11)

where θ is the weighting factor that balances $L_{\mathrm{sim}}$ and $L_{\text{smooth}}$. T denotes the image domain, and ∇(·) represents the spatial gradient operator, while $\vec{u}(p)$ is the displacement vector.

The fusion module is optimized by the designed loss function $L_{\text{fusion}}$. It comprises a reconstruction loss $L_{\mathrm{recon}}$, which computes the absolute difference between IFN output fused images $F_{\mathrm{IFN}}$ and reconstruction constraint-based reference $F_{\mathrm{RC}}$, and a guidance loss $L_{\mathrm{guide}}$, which prevents network confusion and suboptimal results from ambiguous FMEN information constraints. The $L_{\mathrm{fusion}}$ are expressed as follows:^28^

$$L_{\text{fusion}}=L_{\mathrm{recon}}+\rho L_{\text{guide}}={\left\|F_{\mathrm{IFN}}-F_{\mathrm{RC}}\right\|}_{1}+\rho *\frac{1}{2}\overset{2}{\underset{i=1}{\sum }}{\left\|M_{i}-S_{i}\right\|}_{1}\;(i\in \{1,2\}),$$ (12)

where ρ is a weighting parameter. The L1 norm $\|\cdot {\|}_{1}$ is adopted to quantify the discrepancy between the estimated and reference fused images, as well as between the estimated and prior-derived focus maps.

### 2.4 Animals

*In vivo* imaging was performed on a C57BL/6 mouse (body weight: ∼25  g, age: 8 to 10 weeks) provided by the SAHZU Center of Small Animal Experiment, Hangzhou, China (SAHZU: The Second Affiliated Hospital, Zhejiang University School of Medicine). Animal restraint, anesthesia during imaging, and euthanasia after image acquisition were carried out by trained staff at the same facility. All animal procedures followed institutional guidelines for animal care and use and were approved by the Laboratory Animal Welfare and Ethics Review Committee of Zhejiang University (Hangzhou, China), under approval number ZJU20260235.

## 3 Experiment Results

### 3.1 Evaluation Metrics

To quantitatively and comprehensively evaluate the effectiveness of the proposed CRFN, we employed both global and local objective non-reference quality evaluation metrics (NRQMs). Four global NRQMs were utilized, including the Tsallis entropy-based image fusion metric ($Q_{\mathrm{TE}}$),^34^^,^^35^ the gradient-based metric ($Q_{G}$),^34^ the phase congruency-based metric ($Q_{P}$),^34^ and the mean gradient magnitude (${\overset{¯}{G}}_{\mathrm{mag}}$).^36^ These metrics are defined as follows:

$$Q_{\mathrm{TE}}=\frac{I^{q}(I_{1},F_{\mathrm{IFN}})+I^{q}(I_{2},F_{\mathrm{IFN}})}{H^{q}(I_{1})+H^{q}(I_{2})-I^{q}(I_{1},I_{2})},$$ (13)

where $H^{q}(I_{i})$ denotes the Tsallis entropy of $I_{i}(i\in \{1,2\})$. $I^{q}(I_{i},F_{\mathrm{IFN}})$ measures the Tsallis mutual information between $I_{i}$ and $F_{\mathrm{IFN}}$. $I^{q}(I_{1},I_{2})$ quantifies the mutual information between $I_{1}$ and $I_{2}$. The parameter q specifies the order of the quality metric and q≠1.

$$Q_{G}=\frac{Q^{I_{1},F_{\mathrm{IFN}}}\omega^{I_{1}}+Q^{I_{2},F_{\mathrm{IFN}}}\omega^{I_{2}}}{\omega^{I_{1}}+\omega^{I_{2}}},$$ (14)

where $Q^{I_{i},F_{\mathrm{IFN}}}$ denotes the edge information preservation between $I_{i}$ and $F_{\mathrm{IFN}}$, and $\omega^{I_{i}}$ represents the corresponding weighting factor of $I_{i}$.

$$Q_{P}=(P_{\text{phase}}{)}^{\alpha }(P_{\max}{)}^{\beta }(P_{\min}{)}^{\gamma },$$ (15)

where $P_{\text{phase}}$, $P_{\max}$, and $P_{\min}$ denote the phase congruency, maximum, and minimum moments of phase congruency features from the source images, respectively. The exponential parameters α, β, and γ control the relative importance of three components.

$${\overset{¯}{G}}_{\mathrm{mag}}=\frac{1}{mn}\overset{m}{\underset{j=1}{\sum }}\overset{n}{\underset{k=1}{\sum }}\sqrt{{\left[G_{x}(j,k)\right]}^{2}+{\left[G_{y}(j,k)\right]}^{2}},$$ (16)

where $G_{x}(j,k)$ and $G_{y}(j,k)$ denote the horizontal and vertical gradient components, respectively, obtained by convolving the image with the Sobel operators.

In addition, three local NRQMs, namely, the SNR, the contrast-to-noise ratio (CNR), and the equivalent number of looks (ENL), were adopted to evaluate image quality within regions of interest (ROIs).^37^^–^^40^ These metrics are given as follows:

$$\mathrm{SNR}=10\;\log_{10}(\frac{\max(I^{2})}{\sigma^{2}}),$$ (17)

$$\mathrm{CNR}=\frac{1}{R}\overset{R}{\underset{r=1}{\sum }}\;\log_{10}(\frac{\left|\mu_{r}-\mu_{b}\right|}{\sqrt{\sigma_{r}^{2}+\sigma_{b}^{2}}}),$$ (18)

$$\mathrm{ENL}=\frac{1}{H}(\overset{H}{\underset{h=1}{\sum }}\frac{\mu_{h}^{2}}{\sigma_{h}^{2}}),$$ (19)

where I represents the pixel value of the image, and σ represents the standard deviation of the background noise area of the image. $\sigma_{r}$ and $\sigma_{b}$ represent the standard deviation of the ROI and background, respectively, while $\mu_{r}$ and $\mu_{b}$ are their respective mean intensity values. H is the number of homogeneous regions, $\mu_{h}$ and $\sigma_{h}$ represent the mean and standard deviation of all homogeneous ROI.

### 3.2 CRFN-Improved OCT Imaging Performance

To evaluate our proposed framework, both qualitative and quantitative assessments were conducted on multiple groups of specimens, including *ex vivo* porcine eyes, porcine muscles, and chicken wings, along with *in vivo* volumetric imaging performed on a C57BL/6 mouse. For *ex vivo* samples, each dataset comprised 512 A-lines per B-scan, 30 repeated B-scans acquired at each spatial position to improve robustness against noise. For *in vivo* samples, each 3D dataset consisted of 160 B-scans, with each B-scan comprising 512 A-lines per B-scan, without repeated frames.

#### 3.2.1 Evaluations of registration and fusion modules

**Fig. 3:**
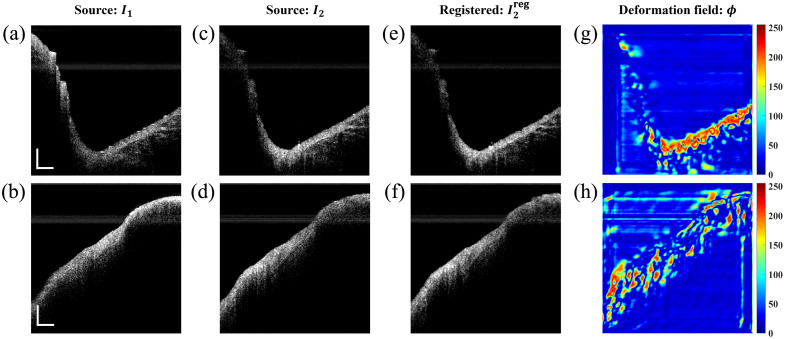
Registration results for representative *ex vivo* chicken wing (top row) and porcine muscle (bottom row). Source B-scan images acquired at focal powers of (a) and (b) +5.75 dpt and (c) and (d) +4.25 dpt are shown. The corresponding (e) and (f) registered images and (g) and (h) estimated deformation fields ϕ are shown. For visualization, each deformation field was normalized by its maximum magnitude and rescaled to the 0 to 255 range. Scale bar: 0.50 mm.

To evaluate the performance of the registration module, two representative B-scan pairs were visualized before and after registration, together with the corresponding estimated deformation fields ϕ, as illustrated in Fig. 3. After registration, the Pearson correlation coefficient^41^ increased from 0.76 to 0.93 for the *ex vivo* chicken wing (top row) and from 0.84 to 0.95 for the *ex vivo* porcine muscle (bottom row). The structural similarity index measure^42^ also increased from 0.54 to 0.82 for the chicken wing and from 0.53 to 0.81 for the porcine muscle. These results demonstrate that the registration module effectively improves image alignment and structural consistency among multi-focus OCT images.

Although the registration loss $L_{\mathrm{reg}}$ stabilized after ∼12,000 iterations, this study primarily focuses on the convergence and performance evolution of the fusion module, as it directly determines the quality of the final fused result. To investigate the evolution of fusion quality during iterative optimization, the quantitative metrics $Q_{\mathrm{TE}}$, $Q_{G}$, and $Q_{P}$ were continuously monitored throughout the process. Two *ex vivo* porcine eye specimens were included in this analysis to provide experimental validation. One specimen comprises only the scleral region, while the other contains three anatomical regions, namely, the cornea, the iris, and the adjacent sclera.

**Fig. 4:**
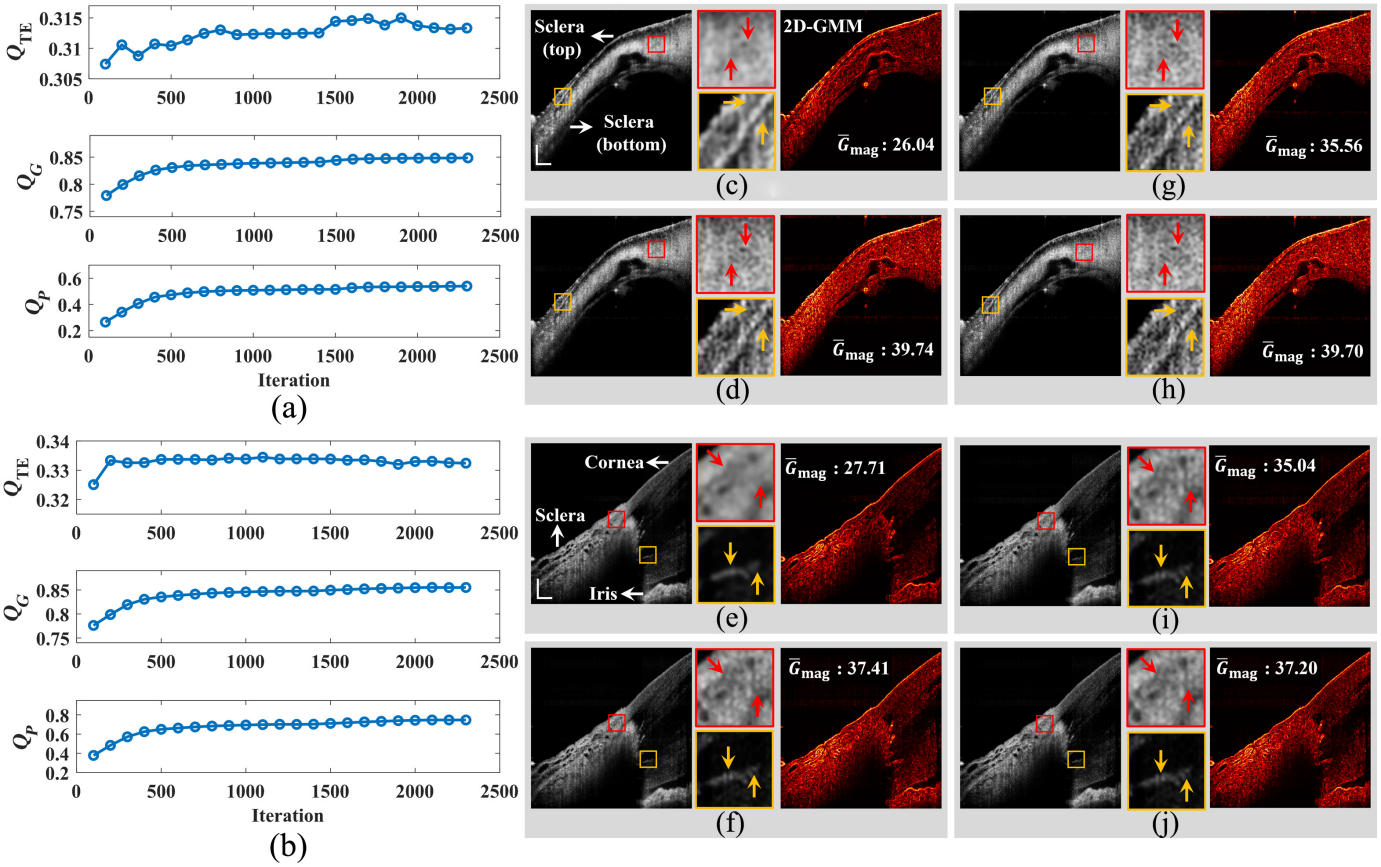
Convergence analysis and evolution of fusion performance in the proposed CRFN. (a) and (b) Evolution of $Q_{\mathrm{TE}}$, $Q_{G}$, and $Q_{P}$ with iteration number for two *ex vivo* porcine eye specimens. (a) Specimen comprising only the scleral region. (b) Specimen containing the cornea, iris, and adjacent sclera. Representative fused images and corresponding 2D Sobel gradient magnitude maps obtained at the (c) and (e) 100th, (g) and (i) 500th, (d) and (f) 1500th, and (h) and (j) 2300th iterations. For each image, the red and orange rectangles indicate the regions selected for magnified visualization, and the corresponding arrows highlight fine structural details. Scale bar: 0.50 mm.

As shown in Figs. 4(a) and 4(b), all three metrics exhibit a substantial rise within the first 500 iterations, indicating a rapid enhancement in fusion quality. From the 500th to the 1500th iterations, the metrics continue to increase more gradually, reflecting steady refinement of the fused results. Beyond ∼1500th iterations, a slight decline is observed in $Q_{\mathrm{TE}}$, suggesting the onset of over-optimization. Representative fused images obtained at the 100th, 500th, 1500th, and 2300th iterations are presented in Figs. 4(c) and 4(e), 4(g) and 4(i), 4(d) and 4(f), and 4(h) and 4(j), respectively. Red and orange rectangles mark enlarged regions, while the corresponding arrows highlight fine structural details. Visually, the fused image at the 500th iteration exhibits pronounced improvements in sharpness and structural continuity relative to the 100th iteration. Although perceptual enhancement from the 500th to 1500th iteration appears less significant, the corresponding two-dimensional (2D) Sobel gradient magnitude maps and their mean gradient values (${\bar{G}}_{\mathrm{mag}}$) still indicate continued gains in image sharpness. After the 1500th iteration, both $Q_{\mathrm{TE}}$ and ${\bar{G}}_{\mathrm{mag}}$ show a slight degradation, corroborating the quantitative trend. Collectively, these findings confirm that CRFN achieves optimal convergence and fusion performance at ∼1500th iteration, which is therefore selected as the representative output for subsequent evaluations.

#### 3.2.2 Qualitative and quantitative evaluations of CRFN-improved results

To evaluate the effectiveness of the proposed CRFN, both qualitative and quantitative comparisons were performed between the fused output and the original source images. B-scan OCT images of the *ex vivo* porcine eye were acquired at two distinct focal planes within the usable focal power range identified in Sec. 2.2.1 (+4 to +6.50  dpt). Specifically, images were acquired at +6.25  dpt for the top of the scleral region (shallow imaging depth) and +4.75  dpt for the bottom of the scleral region (deep imaging depth), providing complementary structural information at opposite ends of the usable focal range. The CRFN-fused output was then directly compared with the corresponding source images to assess improvements in structural preservation, image sharpness, and DOF extension.

**Fig. 5:**
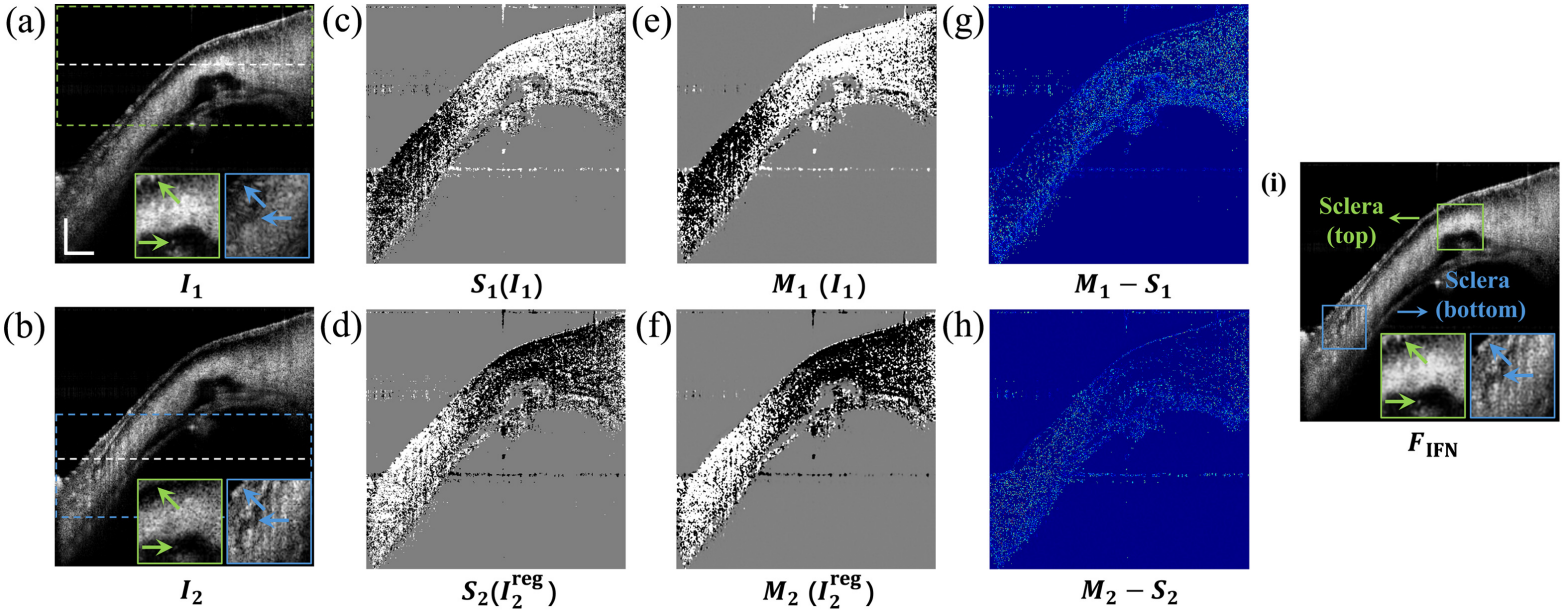
Fusion process and intermediate outputs for the *ex vivo* porcine eye. (a) and (b) Source B-scan images of the *ex vivo* porcine eye acquired at shallower (a) and deeper (b) focal depths. (c) and (d) Prior-generated focus maps ($S_{1}$ and $S_{2}$) corresponding to the source image ($I_{1}$) and the registered image ($I_{2}^{\mathrm{reg}}$). (e) and (f) FMEN output focus maps ($M_{1}$ and $M_{2}$) for $I_{1}$ and $I_{2}^{\mathrm{reg}}$. (g) and (h) Difference maps between FMEN output and prior-generated focus maps. (i) IFN output fused result $F_{\mathrm{IFN}}$. In panels (a) and (b), the white dashed lines indicate the focal plane centers, while the green and blue dashed rectangles denote the DOF of $I_{1}$ and $I_{2}$, respectively. Shallow-focus (green) and deep-focus (blue) ROIs in each image were selected, with magnified views shown at the bottom right. Arrows in magnified views highlight the fine structural details. Scale bar: 0.50 mm.

In the B-scan OCT image with FP of +6.25  dpt ($I_{1}$), higher sensitivity is observed at shallow image depths (∼0 to 1.65 mm), whereas the deeper regions exhibit decreased signal intensity and loss of fine structural detail due to defocusing, as shown in Fig. 5(a). The white dashed line marks the focal plane center, and the green dashed rectangle approximates the DOF region. In practice, optical aberrations such as field curvature cause the actual DOF region to deviate from an ideal rectangular shape, resulting in a more irregular geometry. In contrast, the B-scan OCT image with FP of +4.75  dpt ($I_{2}$) clearly visualizes deeper structures (∼1.45 to 2.85 mm), while the shallower depths suffer from reduced sensitivity and degraded resolution, as shown in Fig. 5(b). The white dashed line indicates the focal plane center, and the blue dashed rectangle outlines the approximate DOF region. Figures 5(c) and 5(d) illustrate the prior-generated focus maps for $I_{1}$ and $I_{2}^{\mathrm{reg}}$, while Figs. 5(e) and 5(f) display the FMEN-estimated focus maps for $I_{1}$ and $I_{2}^{\mathrm{reg}}$, with the difference maps displayed in Figs. 5(g) and 5(h). Compared with the magnified green and blue ROIs in $I_{1}$ and $I_{2}$, the CRFN-enhanced result [Fig. 5(i)] achieves consistently higher sensitivity, effectively suppresses speckle noise, and preserves fine structural details across an extended imaging depth range of ∼0 to 2.85 mm, corresponding to roughly a twofold improvement in effective DOF.

**Fig. 6:**
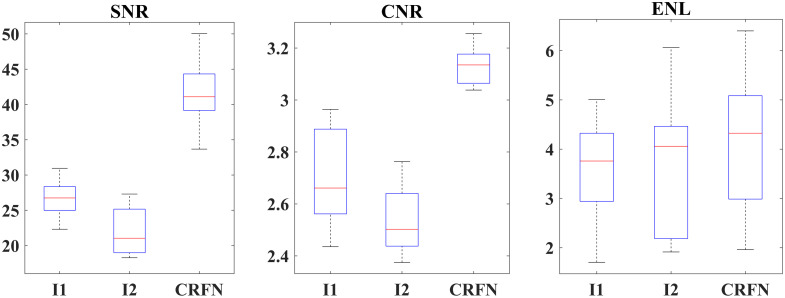
Comparison of SNR, CNR, and ENL between source and CRFN-improved results.

To evaluate the robustness and generalizability of our proposed framework, quantitative evaluation of the CRFN-improved results was performed across multiple specimen types. Three representative ROIs at different depths (shallow, middle, and deep), each ∼0.6  mm×0.6  mm in size, were selected to compute the local NRQMs. Figure 6 presents a comparison of SNR, CNR, and ENL between source and CRFN-improved results, with the corresponding values (mean ± standard deviation) reported in Table 1. Paired t-tests^43^ demonstrate that CRFN-improved results lead to significant improvements in SNR (p-values of $2.60\times 10^{-4}$ for the $I_{1}$ dataset and CRFN-improved images and $3.65\times 10^{-6}$ for the $I_{2}$ dataset and CRFN-improved images) and CNR (p-values of $3.94\times 10^{-4}$ for the $I_{1}$ datasets and CRFN-improved images and $4.37\times 10^{-6}$ for the $I_{2}$ datasets and CRFN-improved images), while ENL shows only a slight increase (with both p-values greater than 0.05).

**Table 1:** Quantitative evaluation of local NRQMs for CRFN-improved results.

Datasets	SNR ↑	CNR ↑	ENL ↑
$I_{1}$	26.68±2.71	2.70±0.19	3.59±1.07
$I_{2}$	21.99±3.49	2.53±0.14	3.67±1.45
CRFN	41.60±4.84	3.13±0.07	4.14±1.46

Arrows indicate the desirable direction for each metric (↑: higher is better), and the best results are highlighted in bold.

#### 3.2.3 Comparison with conventional methods

**Fig. 7:**
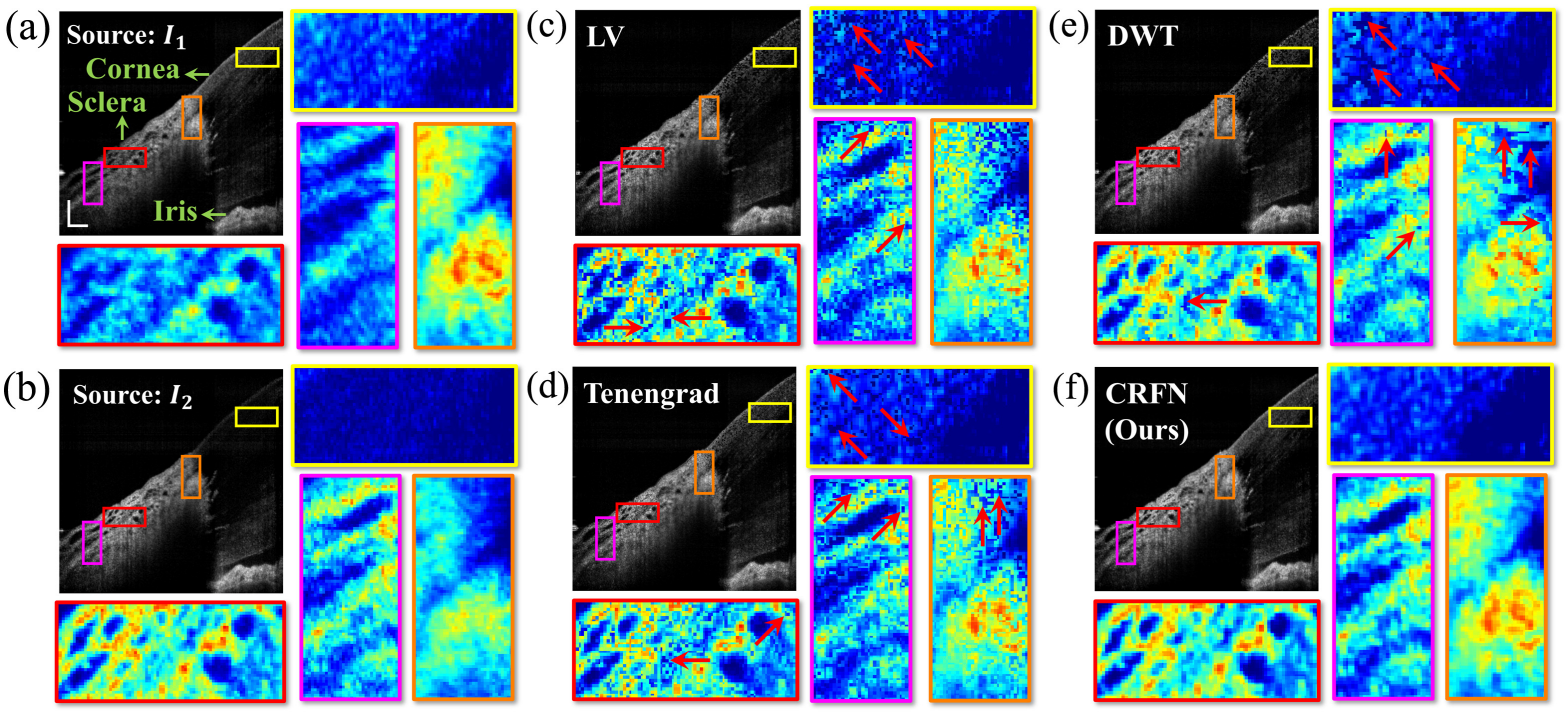
Source images (a) and (b) and fused results obtained by different methods (c)–(f) for the *ex vivo* porcine eye. For clearer visualization, all ROIs in each image are magnified and pseudo-colored, with defect areas appearing as dark-blue points that exhibit strong contrast against surrounding structures. A few representative defect points, rather than all, are indicated by red arrows for illustration. Scale bar: 0.50 mm.

To further demonstrate the superiority of the proposed CRFN, its fused output was compared with results from representative conventional methods, as shown in Fig. 7. Experiments were conducted on the *ex vivo* porcine eye containing the cornea, iris, and the adjacent sclera regions, with two additional focal planes selected within the usable focal power range. This design allows evaluation of CRFN performance across different structural details and focal positions, further demonstrating its robustness and general applicability. Specifically, the image acquired at +5.75  dpt [Fig. 7(a)] enables clear observation of shallow image depths (∼0.20 to 2.20 mm), whereas the image was obtained at +4.25  dpt [Fig. 7(b)] clearly visualizes deeper structures (∼1.95 to 3.50 mm), providing complementary structural information.

Three classical focus-measure-based fusion approaches, consisting of Laplacian variance (LV),^44^ Tenengrad,^45^ and discrete wavelet transform (DWT),^46^ were chosen for comparison. Their fused results (Figs. 7(c)–7(e)] were obtained by weighted averaging of the source images using the corresponding focus maps. All three traditional methods introduce noticeable artifacts manifested as dark spots, which disrupt image smoothness and structural continuity. To better visualize these defects, the ROIs highlighted in Fig. 7 are enlarged and pseudo-colored. In these views, signal loss defects appear as dark-blue points with strong spatial contrast, with representative examples marked by red arrows. The underlying causes vary across algorithms. LV-based fusion estimates focus using local variance, which is highly susceptible to noise. In low-contrast corneal areas and structurally complex scleral regions, the local variance is often underestimated, resulting in attenuated pixel intensities and dark artifacts. Tenengrad-based fusion computes gradient energy using Sobel operators. Weak gradients in low-contrast or complex structural regions lead to inaccurate weights and similar dark-spot defects. DWT-based fusion decomposes images into multi-scale sub-bands, but irregular or noisy high-frequency coefficients in low-contrast regions disrupt texture continuity and introduce local artifacts.

Quantitative evaluations were conducted on the four ROIs (0.5  mm×0.8  mm) in Fig. 7, as summarized in Table 2. The proposed CRFN [Fig. 7(f)] demonstrates the most balanced and consistently strong performance across all NRQMs. These findings show that CRFN excels in edge sharpness, structural consistency, information preservation, and noise suppression.

**Table 2:** Quantitative evaluation of the CRFN-advanced results against conventional and existing DL-based methods, using global and local NRQMs.

Method	Global NRQMs			Local NRQMs		
	$Q_{\mathrm{TE}}$ ↑	$Q_{G}$ ↑	$Q_{P}$ ↑	SNR ↑	CNR ↑	ENL ↑
LV	0.31	0.78	0.32	39.15	2.01	2.86
Tenengrad	0.32	0.80	0.28	39.22	2.67	3.78
DWT	0.32	0.82	0.37	38.81	2.44	3.53
CRFN (ours)	**0.33**	**0.85**	**0.71**	**47.10**	**3.57**	**5.47**

Arrows indicate the desirable direction for each metric (↑: higher is better). The best results are in bold.

#### 3.2.4 In vivo volumetric OCT imaging

**Fig. 8:**
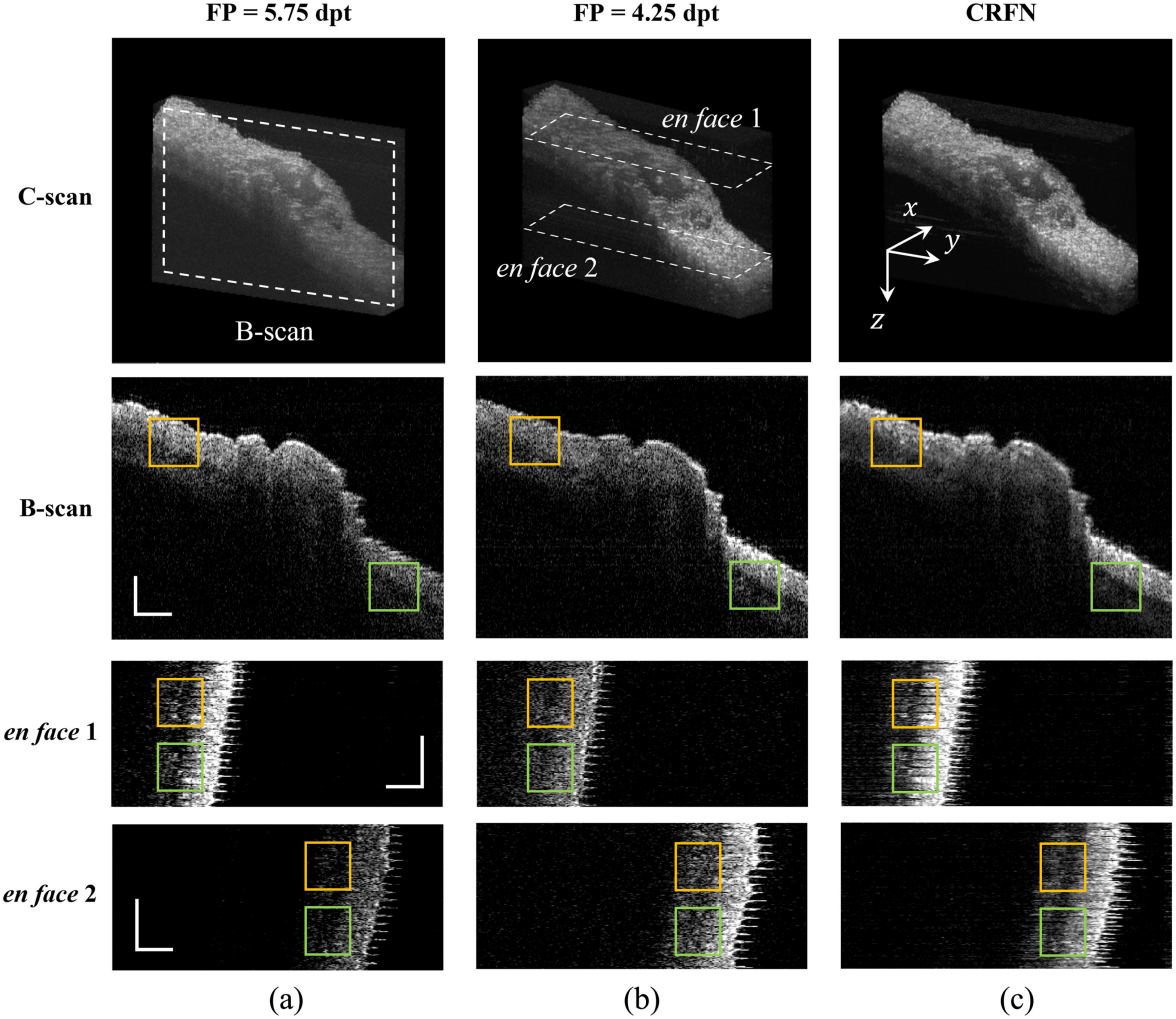
*In vivo* 3D OCT imaging of the mouse chest. (a) and (b) Source images acquired at focal powers of +5.75 (a) and +4.25 dpt (b). (c) CRFN-improved 3D OCT imaging. For each column, row 1 shows the 3D OCT visualization generated using maximum intensity projection, row 2 shows a representative B-scan image, and rows 3 and 4 show *en face* images from shallow and deeper depths, respectively. Scale bar: 0.50 mm.

To evaluate the effectiveness of our proposed framework for *in vivo* volumetric OCT imaging, C-scan OCT image datasets were acquired from a male C57BL/6 mouse (body weight: ∼25  g, age: 8 to 10 weeks) at focal powers of +5.75 and +4.25  dpt, respectively. Figure 8 shows the 3D visualizations using the maximum intensity projection method, B-scan image, along with the shallow and deeper *en face* images. When the focal power is fixed at +5.75  dpt [Fig. 8(a)], it allows clear observation of the upper part of the mouse’s chest, while the lower part remains unclear. When the focal power is adjusted to +4.25  dpt [Fig. 8(b)], it enables clear observation of the lower part of the mouse chest, with the upper part remaining unclear. The results from the shallow and deep *en face* images further support this observation. Figure 8(c) presents the CRFN-fused results, showing improved visualization of both shallow and deeper structures in the mouse chest.

To further quantitatively evaluate the improvement, two ROIs (0.6  mm×0.6  mm) were selected in each of the B-scan images, as well as in the shallow and deeper *en face* images. The results in Table 3 demonstrate that CRFN-fused images show a slight improvement in SNR and CNR, while it is relatively small compared with the *ex vivo* samples. Compared with *ex vivo* samples, the live imaging subjects exhibit motion artifacts, which exacerbate the geometric distortions in the imaging results under two different focal powers, thereby increasing the difficulty for the registration and fusion modules in the framework.

**Table 3:** Quantitative evaluation of local NRQMs for CRFN-improved results.

Images	SNR ↑	CNR ↑	ENL ↑
+5.75 dpt	27.34	2.08	2.34
+4.25 dpt	26.77	2.30	**2.42**
CRFN-fused	**28.75**	**2.63**	2.39

Arrows indicate the desirable direction for each metric (↑: higher is better). The best results are highlighted in bold.

### 3.3 Ablation Study

To systematically assess the contribution of each key component in our proposed CRFN framework, we conducted an ablation study by incrementally removing essential modules, including the difference map-based binary mask $\tilde{B}$ and the registration module.

**Table 4:** Performance comparison of different CRFN variants in the ablation study using global evaluation metrics.

Variant	$Q_{\mathrm{TE}}$ ↑	$Q_{G}$ ↑	$Q_{P}$ ↑
Full CRFN framework	**0.330**	**0.805**	**0.449**
Without guidance loss	0.326	0.804	0.444
Fusion-only	0.312	0.801	0.428

Arrows indicate the desirable direction for each metric (↑: higher is better). The best results are highlighted in bold.

As demonstrated in Table 4, the full CRFN framework achieves the best performance across all metrics, demonstrating the effectiveness of the complete design. When the $\tilde{B}$ is removed, a slight degradation in performance is observed across all global NRQMs, indicating that although separating the sample and background regions helps suppress noise in the original image, it does not directly lead to a significant improvement in fusion quality. In contrast, the fusion-only variant, which omits both the registration module and the binary mask $\overset{˜}{B}$, results in a more significant degradation across all evaluation metrics. This result emphasizes the importance of jointly modeling registration and focus-aware fusion. Without these mechanisms, the ability to recover high-quality fused representations is significantly weakened.

### 3.4 Parameter Sensitivity Analysis

**Fig. 9:**
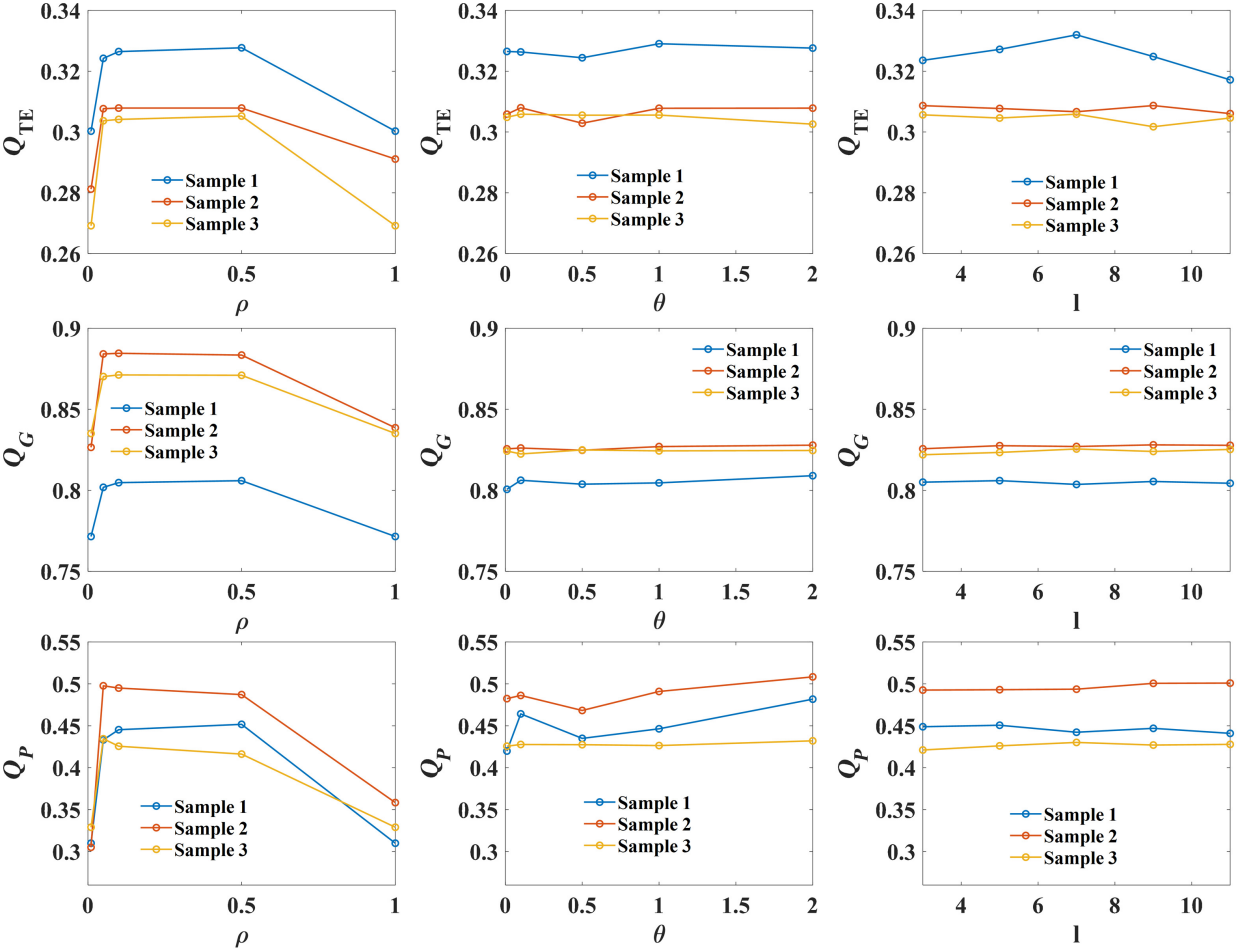
Global NRQMs ($Q_{\mathrm{TE}}$, $Q_{G}$, and $Q_{P}$) for the proposed CRFN with varied weighting parameters (ρ for fusion module and θ for registration module) and window size (l=2r+1).

Figure 9 shows global NRQMs for three representative samples of different values of ρ, θ, and l. The global NRQMs initially increase with ρ, varying relatively smoothly when ρ is between 0.05 and 0.5, but experience a significant decline when ρ increases to 1. This suggests that the guidance loss $L_{\text{guide}}$ effectively guides the focus map estimations and imposes beneficial constraints on the IFN. However, an excessively high $L_{\text{guide}}$ may weaken the reconstruction constraint $L_{\mathrm{recon}}$, leading to a degradation in network performance. The global NRQMs do not exhibit a significant decline over a wide range of smoothness regularization parameter θ values (from 0 to 2), illustrating that our model is robust to the choice of θ. However, it should be noted that excessively large values of θ may cause the registered images to become overly smoothed. The values of $Q_{G}$ and $Q_{S}$ vary relatively smoothly when the window size changes between 3 and 11, while $Q_{\mathrm{TE}}$ starts to decline when the window size exceeds 7. This indicates that a larger window may lead to the loss of image details, thereby affecting the final image quality.

**Fig. 10:**
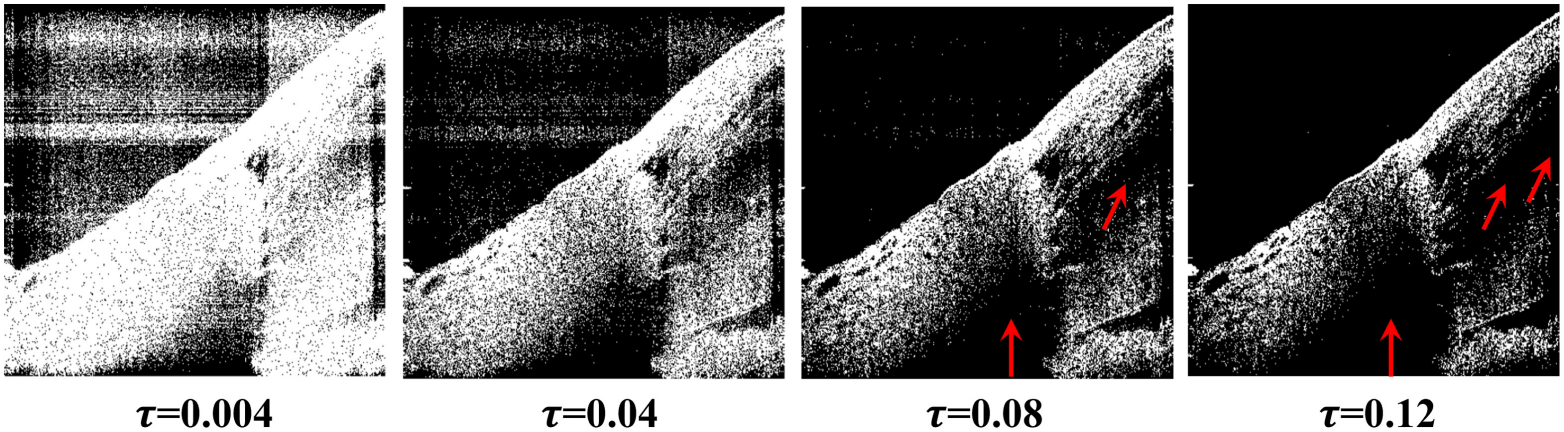
Difference map-based binary mask priors $\tilde{B}$ under different threshold τ values.

The threshold τ for the difference map-based binary mask $\tilde{B}$ needs to be manually selected based on factors such as the sample intensity and background noise intensity in the input normalized images. Figure 10 illustrates the generated binary mask $\tilde{B}$ for a set of *ex vivo* porcine eye samples at various threshold τ values. A larger threshold τ may result in $\tilde{B}$ incorrectly classifying valid sample regions as background, thereby causing the loss of important information (as indicated by the red arrows). In contrast, a smaller threshold may include excessive background information in the mask. Therefore, a balanced value of τ=0.04 is selected for the *ex vivo* porcine eye samples.

### 3.5 Computational Efficiency Analysis

The proposed CRFN was implemented on a workstation equipped with Intel Core i9-13900K CPU, an NVIDIA GeForce RTX 4090 GPU, and 64 GB RAM. The implementation was developed in Python 3.10 on an Ubuntu 22.04 operating system. It was optimized using the Adam optimizer^47^ with an initial learning rate of $5\times 10^{-3}$. A multi-step learning rate decay was applied, reducing the learning rate by a factor of 0.5 at iterations 200, 400, and 800.

**Table 5:** Comparison of computational efficiency between our proposed framework and the conventional registration-weighted fusion-based pipeline.

Method	Process	Iteration	Time (s)	Parameter	Resolution
Traditional	Registration	12,000	381	/	432×432
	Fusion	1	0.04	/	
	Registration + fusion	12,001	381.04	/	
Ours	Registration	12,000	21	0.09 M	
	Fusion	1500	63	4.66 M	
	Registration + fusion	13,500	84	4.75 M	

To evaluate the computational efficiency of our proposed framework, we quantitatively analyze processing time and model complexity, comparing them with those of a conventional registration-weighted fusion-based pipeline. All experiments were conducted using a pair of source images with a resolution of 432×432  pixels to ensure a fair comparison. As summarized in Table 5, the proposed method requires ∼84  s per B-scan, with 21 s for the registration module and 63 s for the fusion module. In comparison, the conventional baseline method requires 381 s for registration and 0.04 s for weighted fusion, resulting in a total processing time of 381.04 s per B-scan. These results demonstrate that our proposed framework significantly reduces computational costs. In terms of model complexity, the registration module contains 0.09 million parameters, and the fusion module contains 4.66 million parameters, yielding a total of 4.75 million parameters. This relatively compact model size is particularly advantageous for OCT applications where computational resources may be limited.

## 4 Discussion and Conclusion

In this paper, we introduced CRFN as a solution to the fundamental challenges inherent in multi-focus OCT imaging, namely, misalignment, structural inconsistencies, and loss of fine details when directly compositing images acquired at different focal planes. Although ETL-based dynamic focus OCT enables efficient acquisition of multi-focus datasets, these hardware strategies alone cannot fully resolve misalignment or guarantee high-fidelity reconstruction. CRFN addresses these limitations through a two-stage architecture: a registration module, comprising a DFPN and an STN, ensures precise spatial alignment, and a fusion module, consisting of a FMEN and an IFN, integrates complementary focus information to produce high-quality fused reconstructions with improved image sharpness and structural continuity.

Despite these advantages, the proposed test-time optimization framework introduces additional computational cost due to its iterative process, which currently limits real-time applications. Several strategies could be adopted to reduce effective processing time without modifying the network architecture. First, the convergence analysis in Fig. 4 shows that the fusion quality metrics tend to stabilize after ∼500 iterations, whereas the current 1500-iteration setting was chosen conservatively to ensure robust convergence and stable reconstruction quality. Therefore,an adaptive early-stopping criterion could be introduced to terminate optimization when the relative change in the loss function remains below a predefined threshold over consecutive iterations. Based on the observed convergence behavior, reducing the fusion stage to ∼500 iterations could provide an estimated threefold speedup. Second, for 3D volumetric OCT datasets, adjacent B-scans typically share similar anatomical structures and intensity distributions. The optimized network weights obtained from the (N)th B-scan could therefore be used as a warm-start initialization for the (N+1)th B-scan, rather than restarting the optimization from random initialization for each slice. This strategy is expected to reduce the number of iterations required for convergence in subsequent slices, thereby amortizing the per-image optimization cost across the volume. Third, mixed-precision optimization could be implemented using standard deep-learning frameworks without changing the network architecture. Compared with the current single-precision (32-bit floating point) implementation, 16-bit floating point computation with appropriate numerical scaling has the potential to reduce memory usage and runtime while maintaining comparable convergence behavior and reconstruction quality.

The proposed framework effectively extends the DOF while preserving fine structural details and image sharpness within the evaluated focal range. However, its performance may degrade under more extreme focal separations, where severe defocus reduces structural visibility and weakens feature correspondence required for reliable registration. In such cases, the optimization process may become less stable and fusion accuracy may be affected. These observations suggest that the current method is most suitable for moderate DOF extension scenarios (∼5  mm in air), while more aggressive extensions remain a potential direction for future improvement.

In conclusion, a self-optimizing and data-efficient framework, CRFN, was developed for multi-focus OCT image registration and fusion. By combining ETL-enabled multi-focus acquisition with a test-time optimization strategy, CRFN extends the effective DOF of OCT imaging and improves reconstruction quality without increasing hardware complexity or requiring large-scale pre-collected training data. The reconstructed images preserve fine structural details and image sharpness within the evaluated focal range, enabling clearer visualization of biological tissue structures. These findings highlight the robustness and practical potential of CRFN for biomedical OCT imaging applications that require extended depth and high-quality structural visualization.

## Supplementary Material

10.1117/1.JBO.31.7.076005.s01

## Data Availability

The implementation code of the proposed framework is publicly available at https://github.com/Endoscope-Lab/CRFN.git. The data underlying the results presented in this paper are not publicly available at present but may be obtained from the authors upon reasonable request.
