# Supplementary material for: Depth-of-focus enhancement in optical coherence tomography via a cascaded image registration and fusion network for multi-focus imaging
Source: J Biomed Opt. 2026 Jul 28;31(7):076005. doi: 10.1117/1.JBO.31.7.076005 (PMC13413486; doi:10.1117/1.JBO.31.7.076005)
Supplement: Supplementary file 1 [file JBO_031_076005_SD001.pdf]

Supplementary Material

ETL-Incorporated SS-OCT System

Fig. S1 presents the customized swept-source optical coherence tomography (SS-OCT) system integrated with an electrically tunable lens (ETL) to achieve dynamic focal plane modulation. The swept-source laser (SSL) has a central wavelength of 1310 nm and a tuning range of 135 nm (AXP50125-6, Excelitas Technologies Crop.). The laser output is coupled into a 1×2 fiber coupler (FC1), which divides the light into the sample (90%) and reference (10%) arms. In the sample arm, the light is guided through an optical circulator (OC) and delivered to a fiber collimator (FCL), which generates a collimated beam of approximately 3 mm in diameter. The beam then passes through an ETL (EL-16-40-TC-NIR-20D, Optotune Inc.), which enables millisecond-level focal plane modulation. Subsequently, the beam is directed through a dual-axis galvanometer scanner (DGS) and an achromatic doublet (AD), enabling two-dimensional (2D) raster scanning and focusing on the sample. The FCL, ETL, DGS, and AD are integrated into a compact optical assembly, as highlighted by the red rectangular region in Fig. S1. In the reference arm, a variable optical delay line (ODL) is used to maintain path length matching. The backscattered light from the specimen is returned through the OC and interferes with the reference beam in a 2×2 fiber coupler (FC2). The resulting interference signals are detected using a balanced photodetector (BPD) and subsequently digitized by a data acquisition (DAQ) card (ATS9352, AlazarTech Inc.).

Precise synchronization among SSL, DGS, and DAQ was achieved through programmed timing control. As illustrated by black dash line in Fig. 1, the laser trigger signal (100 kHz) is split into two channels: one (*TRI1*) drives a function generator (FG) to produce the control signals for the x- and y-axes (*A1* and *A2*) within the DGS, while the other (*TRI2*) serves as an external trigger for the DAQ. Additionally, a digital gating signal (*DIG*) generated from FG is delivered to the DAQ AUX I/O port to enable gated acquisition during effective forward scanning. The laser k-clock signal (*KCL*) is used as a sampling external clock to ensure wavenumber-domain linearization.

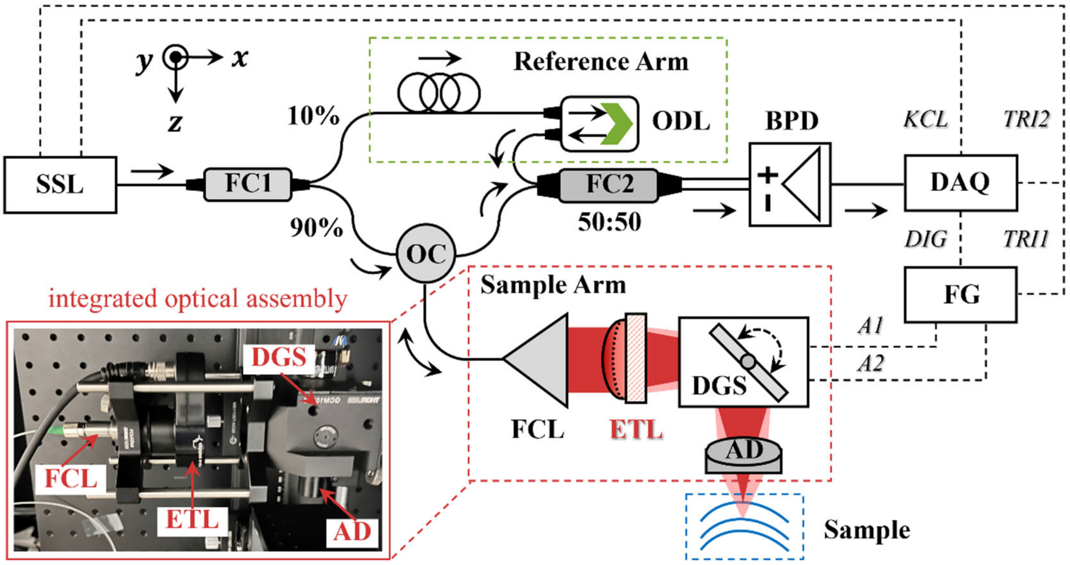

Fig. S1 Schematic of the ETL-incorporated SS-OCT system for dynamic focal plane modulation.

Chromatic Aberration Analysis across the Swept-Source Bandwidth

Fig. S2 demonstrates that the measured full width at half maximum (FWHM) of the axial point spread function (PSF) at a focal power of +6.5 dpt exhibits negligible difference between the two cases (17.66 μm vs. 18.40 μm), indicating no measurable degradation of axial resolution introduced by the ETL within the operating wavelength range.

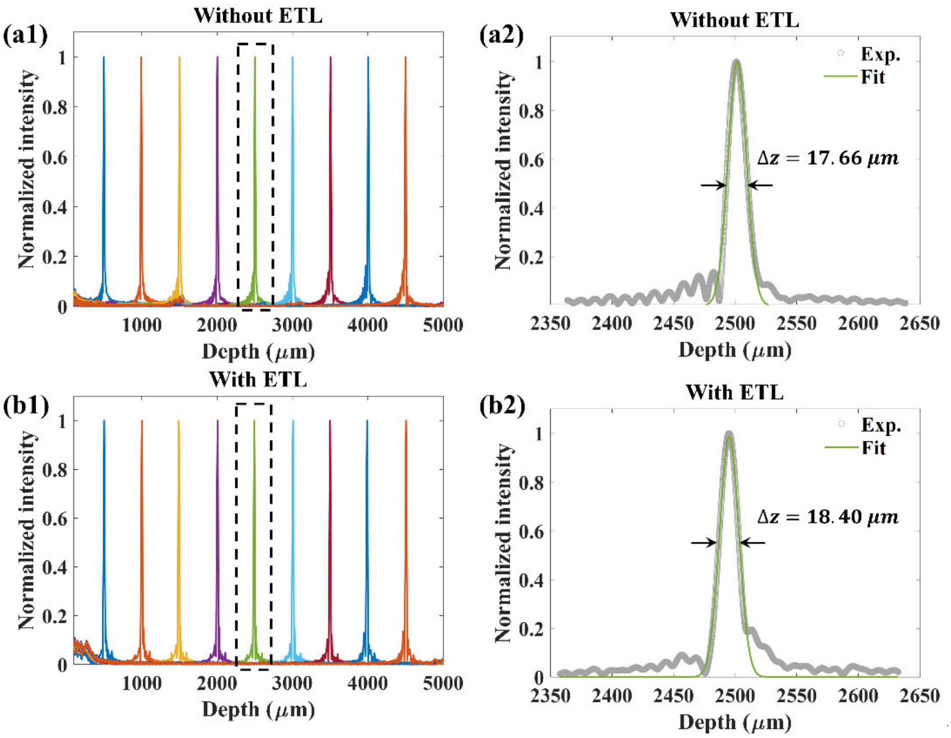

Fig. S2 Normalized depth-dependent axial resolution without (a1, a2) and with (b1, b2) the ETL in the optical path. The magnified views (a2, b2) show the axial PSF at ~2.5 mm depth, with black dash dashed lines indicated in (a1, b1).

Focal Power Reproducibility of ETL

Fig. S3 displays boxplots of axial peak position variations across four representative depths, showing narrow interquartile ranges (IQRs) of 1.37, 2.38, 1.83, and 2.10 μm, with standard deviations (SDs) below 1.39 μm. These variations are significantly smaller than the system axial resolution, indicating stable focal positioning and high reproducibility of the ETL across repeated acquisitions.

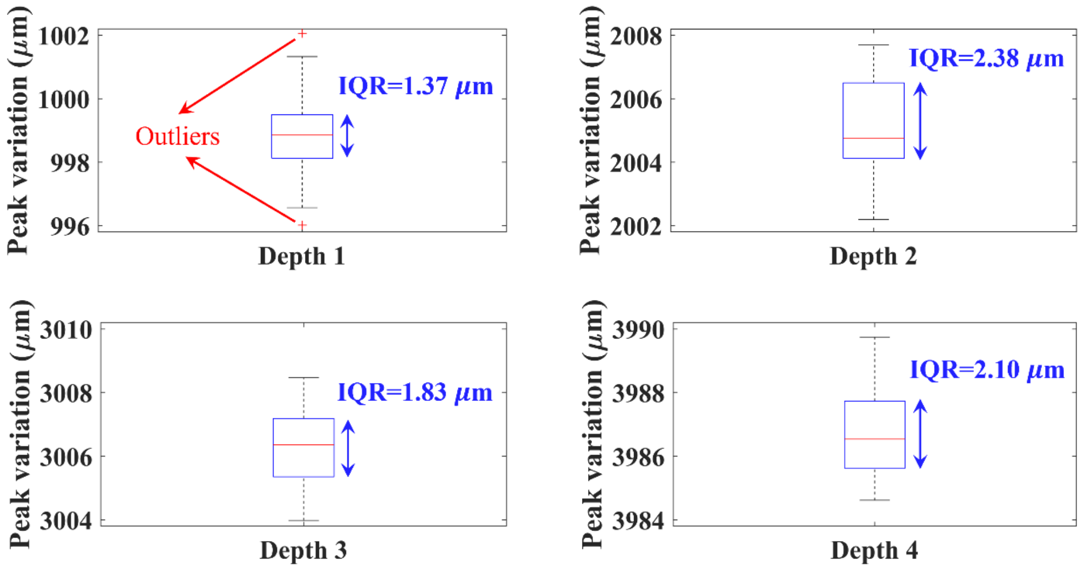

Fig. S3 Boxplots of axial peak position variations at four representative imaging depths under repeated B-scan acquisitions (N = 50) at a focal power of +6.5 dpt.

Axial Optical Path Difference Shift Induced by ETL Focal Tuning

Fig. S4 represents the measured axial optical path difference (OPD) shifts induced by focal tuning from +4.0 to +6.5 dpt at two representative depths. The focal power tuning induces axial OPD shifts of approximately 99.43 μm and 104.10 μm at ~1.5 mm and ~3.5 mm depths, respectively.

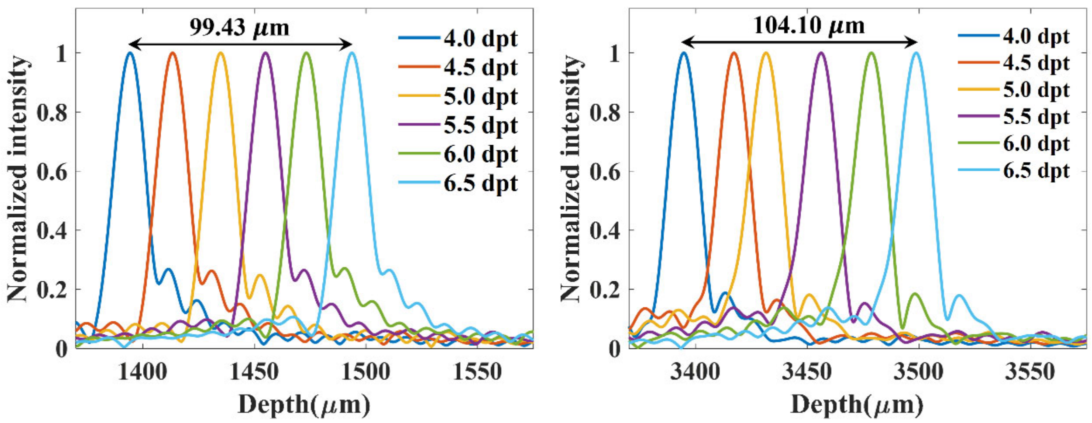

Fig. S4 Measured axial OPD shifts induced by focal tuning from +4.0 to +6.5 dpt at two representative depths (~1.5 mm and ~3.5 mm).
